# ﻿Three Loxocaudinae species (Ostracoda, Podocopida) from South Korea

**DOI:** 10.3897/zookeys.1138.96201

**Published:** 2023-01-06

**Authors:** Hyunsu Yoo, Pham Thi Minh Huyen, Jinho Chae, Ivana Karanovic

**Affiliations:** 1 Marine Environmental Research and Information Laboratory (MERIL), 17, Gosan-ro, 148 beon-gil, Gunpo-si, Gyoenggi-do, 15180, Republic of Korea; 2 Department of Applied Chemistry and Biological Engineering, Graduate School Department of Molecular Science and Technology, Ajou University, Suwon, 16499, Republic of Korea; 3 Department of Life Science, College of Natural Sciences, Hanyang University, Seoul, 04763, Republic of Korea; 4 Research Institute for Convergence of Basic Science, Hanyang University, Seoul 04763, Republic of Korea

**Keywords:** Biodiversity, Cytheroidea, Loxoconchidae, phylogeny, taxonomy

## Abstract

For many ostracod groups in Korea, published records are missing or are very limited. Loxocaudinae is one such subfamily, with only one named species, *Loxocaudaorientalis* Schornikov, 2011 reported from Korea. Having fewer than 50 species, this subfamily can be considered a small ostracod group, with most of the species known only by their shell morphology. The diagnoses of genera are based on the shell characters that are often homoplastic, and soft body appendages that are difficult to observe, such as the mandibular exopodite. Because of this, the validity of the entire subfamily and some of its genera have been questioned. Here three Loxocaudinae species were collected from the marine macrobenthic assemblages from Korea. Two are new and belong to the genus *Glacioloxoconcha* Hartmann, 1990, previously known only from Antarctica: *Glacioloxoconchajeongokensis***sp. nov.** and *Glacioloxoconchajisepoensis***sp. nov.***Loxocaudaorientalis* is briefly redescribed, with some of the populations having unusual morphological features. COI and 18S rRNA sequences of all three species are provided and the latter marker used to assess the position of the subfamily within the family Loxoconchidae and the superfamily Cytheroidea. The resulting tree shows that within the family Loxoconchidae, the genera *Glacioloxoconcha* and *Loxocauda* Schornikov, 1969 are the most closely related, with very shallow but well-supported branches. Polyphyletic and paraphyletic natures of several Cytheroidea families are discussed, inferred from the reconstructed phylogeny.

## ﻿Introduction

The subfamily Loxocaudinae was established by Schornikov (2011a) to encompass the following five genera: *Glacioloxoconcha* Hartmann, 1990; *Loxocauda* Schornikov, 1969; *Phlyctocythere* Keij, 1958; *Pseudoloxoconcha* Müller, 1894, and *Sarmatina* Stancheva, 1984. It is a relatively small group of ostracods, currently accounting for 34 described species, of which 20 belong to *Phlyctocythere*, ten to *Loxocauda*, two to *Pseudoloxoconcha*, and one each to *Glacioloxoconcha* and *Sarmatina*. Their most noticeable morphological characteristics are a prominent caudal process on the shell, an adont hinge, a compact naupliar eye (without eye tubercles), and a smooth shell (with a reduced lateral sculpture). The majority of species were described based on their shells only, and little is known about the soft parts morphology. Soft parts have been described only for one species of *Glacioloxoconcha*, four species of *Loxocauda*, and one each species for *Pseudoloxococnha* and *Phlyctocythere*. Consequently, some authors doubt the validity of a few genera, and the current systematic position of a number of species (Ikeya and Hanai 1982; Schornikov 2011a). Besides the lack of information regarding the soft parts morphology, the reason is also a high similarity in the shell morphology between species currently belonging to different Loxocaudinae genera.

The subfamily Loxocaudinae has a worldwide distribution and species inhabit marine and brackish waters (Brandão and Karanovic 2022). In South Korea, the subfamily is represented by 11 species (Schornikov 2011a), most of which are left in the open nomenclature. Only one of them, *L.orientalis* Schornikov, 2011, was described from the sea grass beds and it seems to be distributed across the northern part of the Far East region (Schornikov 2011b). Here we report two new *Glacioloxoconcha*, and briefly redescribe *L.orientalis*. The genus *Glacioloxoconcha* was originally described from Antarctica (Hartmann 1990) to include *G.suedshetlandensis* Hartmann, 1990, a small phytal species with a conspicuous morphology. In this species all the claws on appendages are weak, segments of antennula and antenna are slender, and male copulatory organ has strong frontal chitinous braces. The new Korean species have also been collected from algal and macrobenthic assemblages. We provide 18S rRNA and CO1 gene sequence data for the two new *Glacioloxoconcha* and *L.orientalis*. The aims of this paper are to provide additional details of the soft body morphology of Loxocaudinae, and reconstruct its phylogenetic position within Cytheroidea. As a result of the phylogenetic reconstruction, we briefly discuss the systematics of the superfamily Cytheroidea.

## ﻿Materials and methods

### ﻿Sampling methods and taxonomy

**Figure 1.**
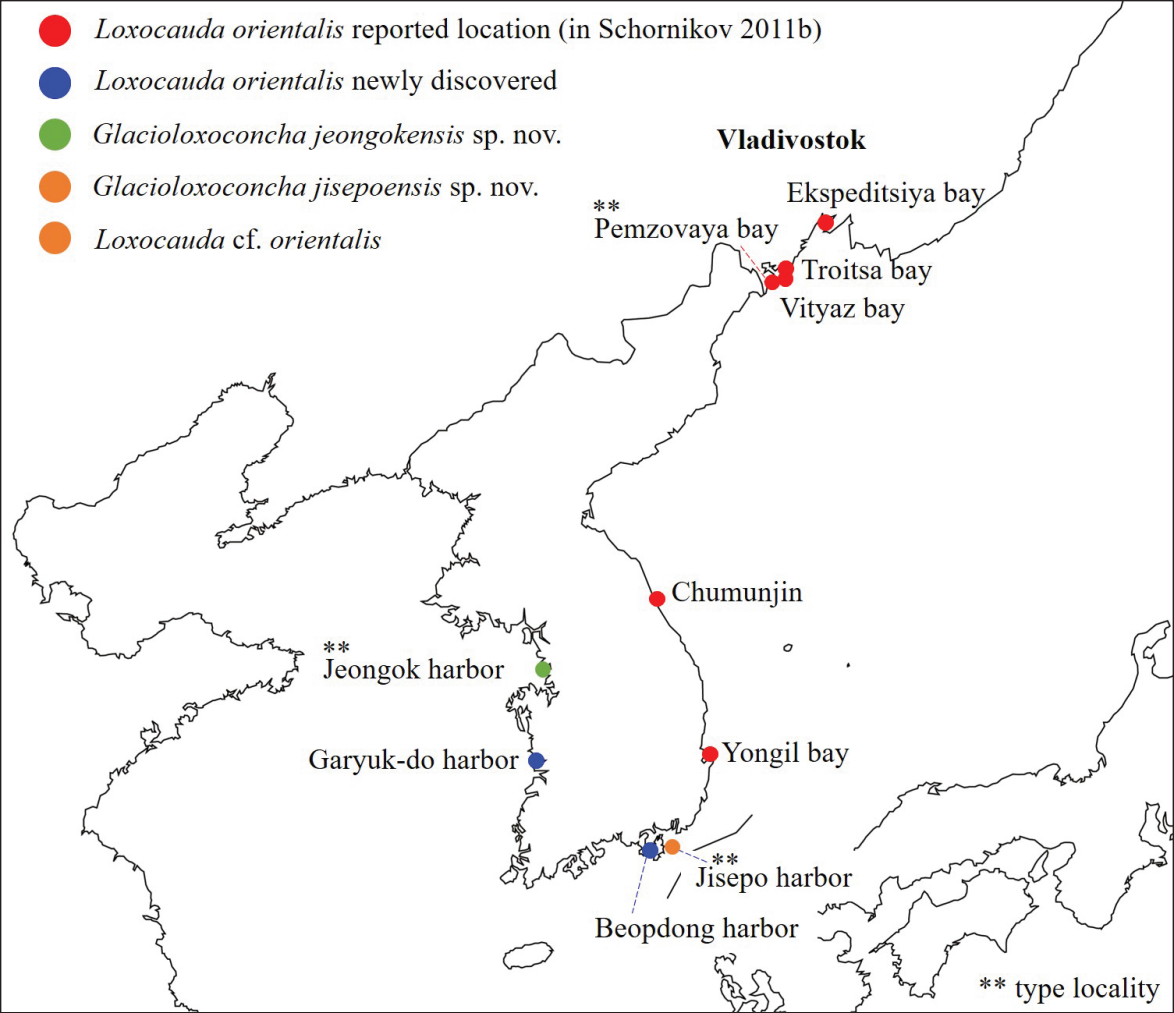
Map of sampling locations from Schornikov (2011b) and the newly collected samples.

**Figure 2.**
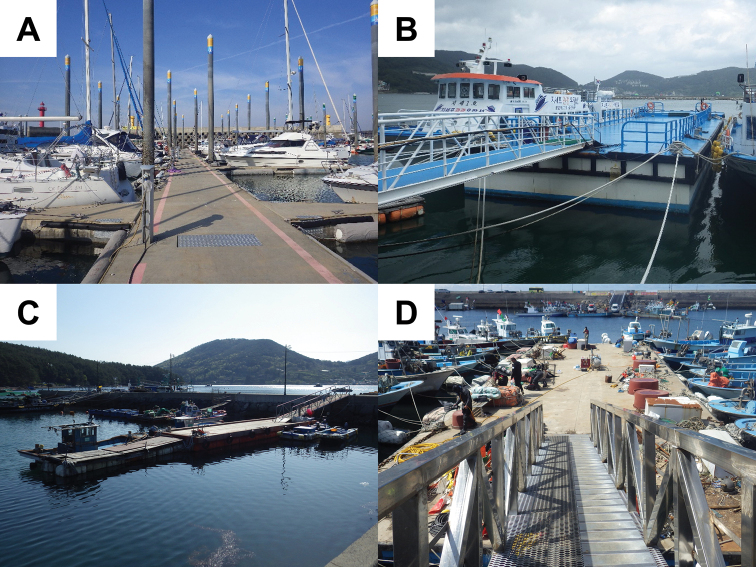
Photographs of the mooring structure near various harbors **A** Jeongok harbor **B** Jisepo harbor **C** Beopdong harbor **D** Garyuk-do harbor.

**Figure 3.**
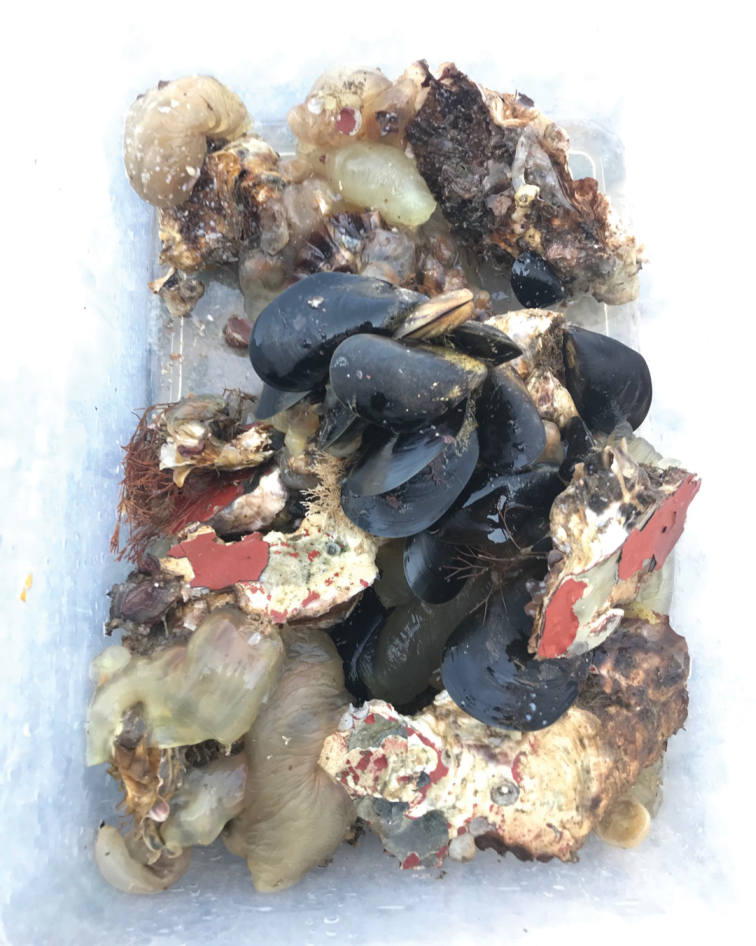
Photographs of a macrobenthos sample including algae (after rinsing).

Macrobenthos attached to boat moorings were initially collected by scuba diving. When brought ashore it was washed and rinsed through a hand-net (mesh size is 63 µm) (Figs 1–3), and directly fixed in 99% ethanol on site. Sorting and dissecting were done under a stereomicroscope (Olympus SZX12) in the laboratory at Hanyang University. Soft parts are first used for the DNA extraction and after that dissected and mounted on slides in the CMC-10 Mounting Media (Masters Company, Inc.). The valves were mounted on SEM stubs and latter stored on micropaleontological slides. All drawings were prepared using a drawing tube, attached to the microscope ZEISS Axioskop 50. For observations under the scanning electron microscope (SEM), carapaces were coated with platinum. SEM photographs were taken at the National Institute of Biological Resources (**NIBR**) and at Hanyang University with JEOL JSM-6390 and COXEM EM-30 electron microscopes. All specimens are deposited either in the collections of the National Institute of Biological Research (**NIBR**) or in the National Marine Biodiversity Institute of Korea (**MABIK**).

### ﻿DNA extraction and molecular data analysis

The extraction followed the HotSHOT method described in Pham et al. (2021). PCR reactions for 18S rRNA gene were carried out in 25 μl volume containing: 5 μl of diluted DNA template, 1 μl of 10 pmol/μl forward and reverse primers, 15 μl free RNA&DNA water and 5 μl AccuPower PCR premix (Bioneer Inc.). COI gene amplification reactions were carried out in 21 μl: 10 μl HotStar Taq mastermix (Qiagen), 5 μl water, 1 μl of each primer at 10 pmol/μl and 2 μl DNA template. Primers used in this study along with the PCR settings are listed in Suppl. material 1. COI primers were designed with the webtool PrimerDesign-M following Brodin et al. (2013) and Yoon and Leitner (2015). PCR products were electrophoresed (for 20 min at 100 V) on 1% agarose gels (0.5X TAE buffer dyed with GelRed Nucleic Acid Gel Stain) to determine the presence of target DNA bands. PCR products were purified for sequencing by ethanol precipitation and neutralized by sodium acetate (pH 5.5). Sequencing reactions were run for both strands to confirm sequence reliability using the Sanger method for dideoxy sequencing (Macrogen Inc. and Bionic Inc., Seoul, South Korea). All obtained sequences have been deposited in GenBank (Suppl. material 2).

Phylogenetic trees were constructed based on the alignment of 18S rRNA and COI genes. For 18S tree, in addition to the newly obtained sequences, we also included 47 sequences belonging to the sub-order Cytherocopina Baird, 1850 deposited on GenBank. Of all available sequences attributed to Cytherocopina we only used those that belong to individuals identified to the species level (see Suppl. material 2). We have chosen *Terrestricytherepratensis* Schornikov, 1980, as the outgroup to root the 18S tree, and *Krithekamchatkaensis* Yoo, Tanaka, Lee, Brandão & Karanovic, 2019 as the outgroup to root the COI tree. Uncorrected p-distances between sequences were calculated in MEGA 7 (Kumar et al. 2016). The best fit evolutionary model was calculated based on the Akaike Information Criterion (AIC) as implemented in ModelFinder (Kalyaanamoorthy et al. 2017). Bayesian Inference, implemented in BEAST v. 2.6.4 (Bouckaert et al. 2014), was used to estimate phylogenetic relationships. Settings included the best fit evolutionary model with four gamma categories and a strict molecular clock. The analysis run for 10,000,000 generations, sampling every 1,000 generations. Tracer v. 1.7.1 (Rambautet et al. 2014) was used to visualize the results of the analyses. The final phylogenetic trees were rooted and visualized by FigTree v. 1.4.3 (Rambaut 2010).

### ﻿Abbreviations used in text and figures:

**A1** Antennula;

**A2** Antenna;

**BO** Brushed organ;

**GF** Genital field;

**H** Height;

**Hp** Hemipenis;

**L** Length;

**LV** Left valve;

**L5–7** Leg 5–7;

**Md** Mandibula;

**Mxl** Maxillula;

**RV** Right valve.

## ﻿Results

### ﻿Systematics

**Order Podocopida Sars, 1866**

**Family Loxoconchidae Sars, 1925**

**Subfamily Loxocaudinae Schornikov, 2011**

#### Genus *Glacioloxoconcha* Hartmann, 1990

##### ﻿ Glacioloxoconcha jeongokensis sp. nov.

Taxon classificationAnimaliaPodocopidaLoxoconchidae

DD0712B0-0032-505A-8525-E205FBEAA370

https://zoobank.org/E7DC6CA1-4CBE-4BCF-8735-2A5C3E3B3372

Figs 4 , 5 , 6

###### Material examined.

***Holotype***, male, dissected on one slide (NIBRIV0000882303) and shell on micropaleontological slide (NIBRIV0000882313); ***Allotype***, female, dissected on one slide (NIBRIV0000882309) and shell on micropaleontological slide (NIBRIV0000882311); ***Paratypes***: one male and one female dissected on each slide, and shell on micropaleontological slides; ~ 20 specimens kept in 2 ml vial in 99% alcohol.

###### Type locality.

South Korea, Gyeonggi-do, Hwaseong-si, Seosin-myeon, Jeongokhang-ro, Yacht mooring. 37°11.179'N, 126°39.024'E, 25 October 2019, leg. Hyunsu Yoo & Byung-jin Yoo.

###### Etymology.

The species is named after the yacht mooring place from where it was collected.

###### Description.

**Figure 4.**
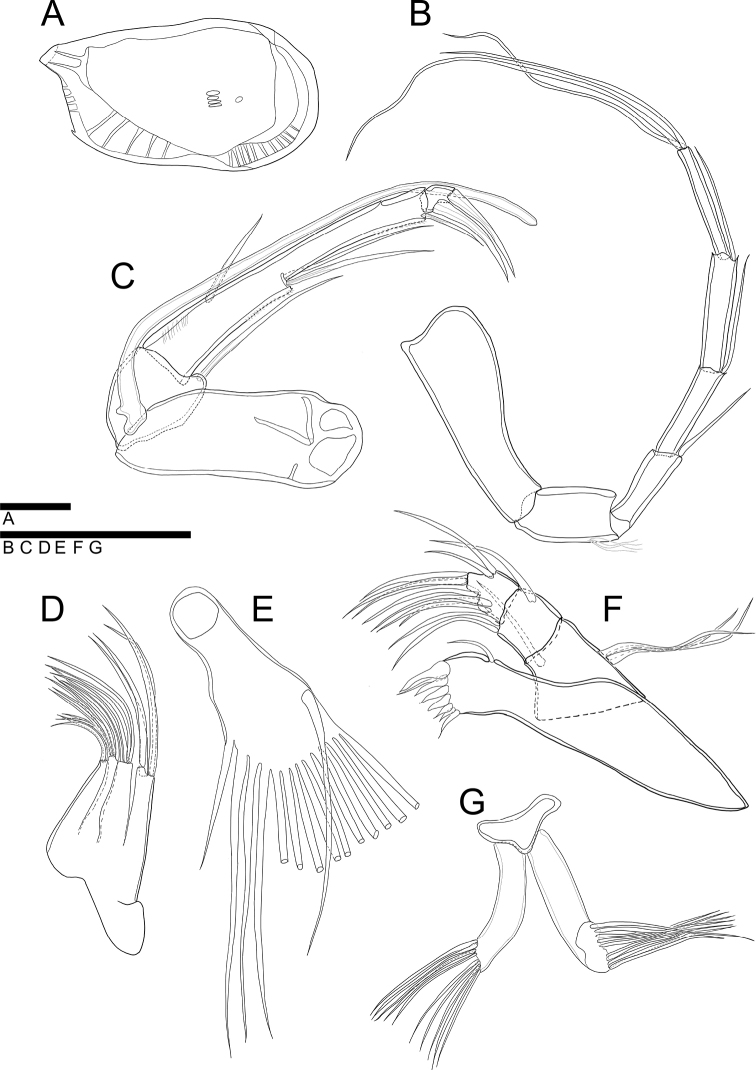
*Glacioloxoconchajeongokensis* sp. nov.: male (NIBRIV0000882303, NIBRIV0000882313 holotype) **A**LV internal view **B**A1**C**A2**D** endopodite of Mxl**E** vibratory organ **F**Md**G**BO. Scale bars: 50 µm (**B–F**); 100 µm (**A**).

**Male. *Carapace*** (Figs 4A, 6). Relatively small, L ~ 356 µm, H ~ 189 µm. RV overlapping LV dorsally. Carapace subquadrate form in lateral view (Figs 4A, 6A). Anterior margin rounded; dorsal margin straight; postero-dorsal margin with extended caudal process; ventral margin almost straight and inclined gently toward posterior margin; postero-ventral margin with two small spines (Figs 4A, 6A, F). Postero-ventral and anterior margins strongly compressed (Fig. 6A). Greatest H situated in front of middle. Eye present. Surface ornamentation consisting of shallow reticulation on postero-dorsal margin, with few simple setae; few sieve-like pores also present (Fig. 6A, B). Marginal pore canals distributed on antero-ventral and posterior margins (Fig. 4A). Posterior inner lamella wider than anterior. Muscular scar imprints consisting of a row of four vertical scars and one frontal scar. Hinge adont (Fig. 4A).

***A1*** (Fig. 4B). Six-segmented. First segment without setulae and setae. Second segment with setulae on antero-distal margin. Third segment with one bare seta antero-distally, reaching end of fourth segment. Fourth and fifth segments each with one bare seta on anterior-distal margin, reaching end of next segment. Terminal segment with three long bare setae on distal margin, almost 2.5 × longer than terminal segment. L ratios between six segments 2.5: 1.1: 1: 1.2: 1.5: 1.4.

***A2*** (Fig. 4C). Four-segmented. Exopod transformed into spinneret seta. First endopodal segment without setulae and seta. Second segment with one bare seta postero-distally reaching 2/3 length of third segment. Third segment with setulae on antero-proximal, postero-medial, and postero-distal margins, and with one bare seta on antero-proximal margin, reaching 1/2 length of the same segment; two bare setae postero-medially, reaching end of the same segment; one bare seta postero-distally, almost 2 × longer than terminal segment. Terminal segment with two strong, bare claws on distal margin almost 3× longer than the same segment. L ratios between four segments 8.3: 3: 11.3: 1.

***Md*** (Fig. 4F). Coxa with seven strong teeth and one thin, bare seta on distal margin, and one bare seta near anterior-distal margin. Exopod with three bare setae; endopod three-segmented. First endopodal segment with one bare seta antero-distally. Second segment with two bare setae antero-distally and one bare seta postero-distally. Terminal segment with nine setae, four of which arise from anterior margin, two from distal margin, and two from postero-distal margin. First segment almost 2× longer than terminal segment.

***Mxl*** (Fig. 4D, E). Palp present. Two-segmented. Terminal segment with four bare setae distally. Exopodite with 1 reflexed seta and ~ 14 bare setae on branchial plate. Masticatory process with three endites, first and second endites each with four bare setae, third endite with two bare setae.

**Figure 5.**
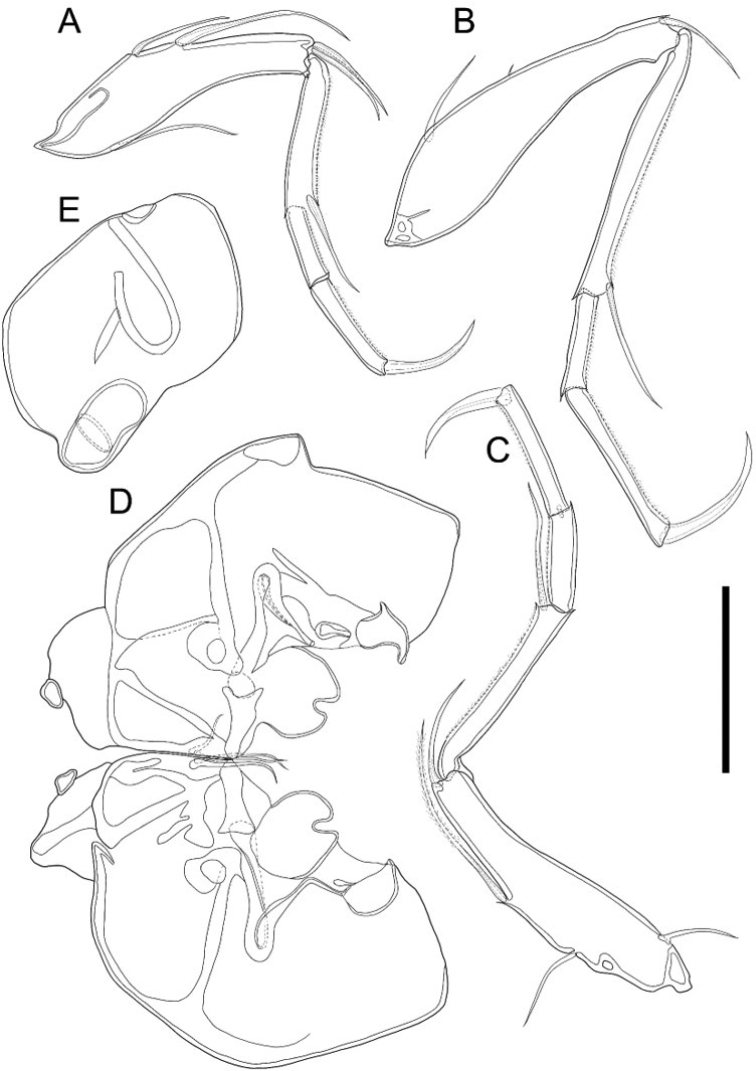
*Glacioloxoconchajeongokensis* sp. nov.: male (NIBRIV0000882303 holotype) **A**L5**B**L6**C**L7**D**Hp; female (NIBRIV0000882309 allotype) **E**GF. Scale bar: 50 µm.

***L5*** (Fig. 5A). Four-segmented. First segment with five bare setae, two antero-medially, one reaching and one not reaching end of the same segment; and two setae antero-distally, reaching 1/2 of second segment; and one postero-proximally, reaching 2/3 length of the same segment. Second segment with one bare seta antero-distally, reaching 1/3 length of the terminal segment. Penultimate segment without any seta. Terminal segment with one claw like seta on distal margin. Last three segments with setulae along anterior margin. L ratios between four segments 3.5: 1.9: 1: 1.4.

**Figure 6.**
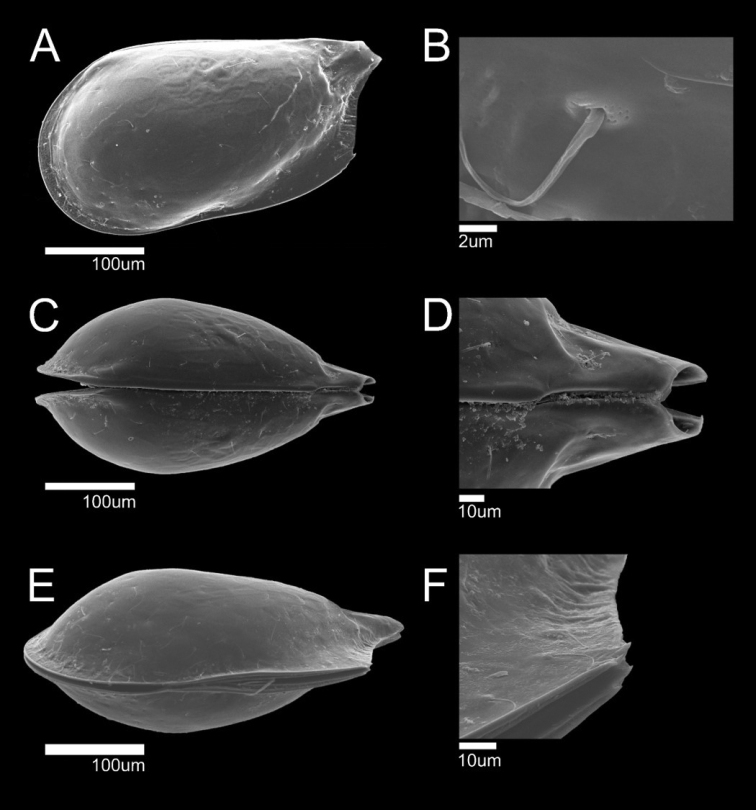
SEM photographs of *Glacioloxoconchajeongokensis* sp. nov.: male (NIBRIV0000882313 holotype) **A**LV external view **B** Simple seta pore on external carapace; male (paratype) **C** dorsal view **D** posterior part of dorsal margin **E** ventral view **F** postero-ventral part of ventral margin.

***L6*** (Fig. 5B). Four-segmented. First segment with three bare setae, one antero-proximally, reaching 1/2 length of the same segment; one tiny seta; and one antero-distally, reaching 1/5 length of second segment. Second segment with one bare seta antero-distally, reaching 1/3 length of terminal segment. Penultimate segment without any seta. Terminal segment with one claw-like seta on distal margin. Last three segments with setulae along anterior distal margin. L ratios between four segments 3.5: 2.8: 1: 1.8.

***L7*** (Fig. 5C). Four-segmented. First segment with four setae: one bare seta posterior-proximally, as long as 1/4 of the same segment; one bare seta on antero-proximally, as long as 1/4 of the same segment; one plumose seta antero-medially, reaching 1/4 length of second segment; one bare seta antero-distally, reaching 1/2 length of second segment. Second segment with one bare seta on anterior-distal margin, reaching 1/3 length of third segment. Third segment without seta. Terminal segment with one strong claw on distal margin, 1/2 as long as the segment. Last three segments with setulae along anterior margin. L ratios between four segments 3.2: 2: 1: 1.3. Compared with L5 and L6 segments, L7 is more elongated than L5, but similar to L6.

***BO*** (Fig. 4G) With more than ten setae on distal margin. Positioned behind L7 and below Hp.

***Hp*** (Fig. 5D). Basal plate sub-rectangular. Lobe rudimentary, shaped as a lotus leaf. CR fused with Hp and represented with two setae.

**Female. *Carapace*** (Fig. 13A). Slightly larger than males. L ~ 382 µm, H ~ 211 µm. Shape and all other morphological features similar to male.

***GF*** (Fig. 5E). Basal part rectangular. CR setae not observed. Ovary sub-rectangular.

All other appendages same as in male.

##### ﻿ Glacioloxoconcha jisepoensis sp. nov.

Taxon classificationAnimaliaPodocopidaLoxoconchidae

1C2DCC46-B1AD-59E6-8BB3-B65344FA77A4

https://zoobank.org/FF235D4C-AEC4-4632-A800-53CA49E9CF10

Figs 7 , 8

###### Material examined.

***Holotype***, male, dissected on one slide (MABIKCR0025819); ***Allotype***, female, dissected on one slide (MABIKCR0025820); ***Paratypes***: one male and female dissected on each slide, and shell on each micropaleontological slide and 5 specimens kept in 2 ml vial.

###### Type locality.

South Korea, Gyeongsangnam-do, Geoje-si, Irun-myeon, Jisepohaean-ro, Jisepo harbor. 34°49.919'N, 128°42.220'E, 19 May 2020, leg. Hyunsu Yoo & Byung-jin Yoo.

###### Etymology.

The species is named after the harbor from where it was collected.

###### Description.

**Figure 7.**
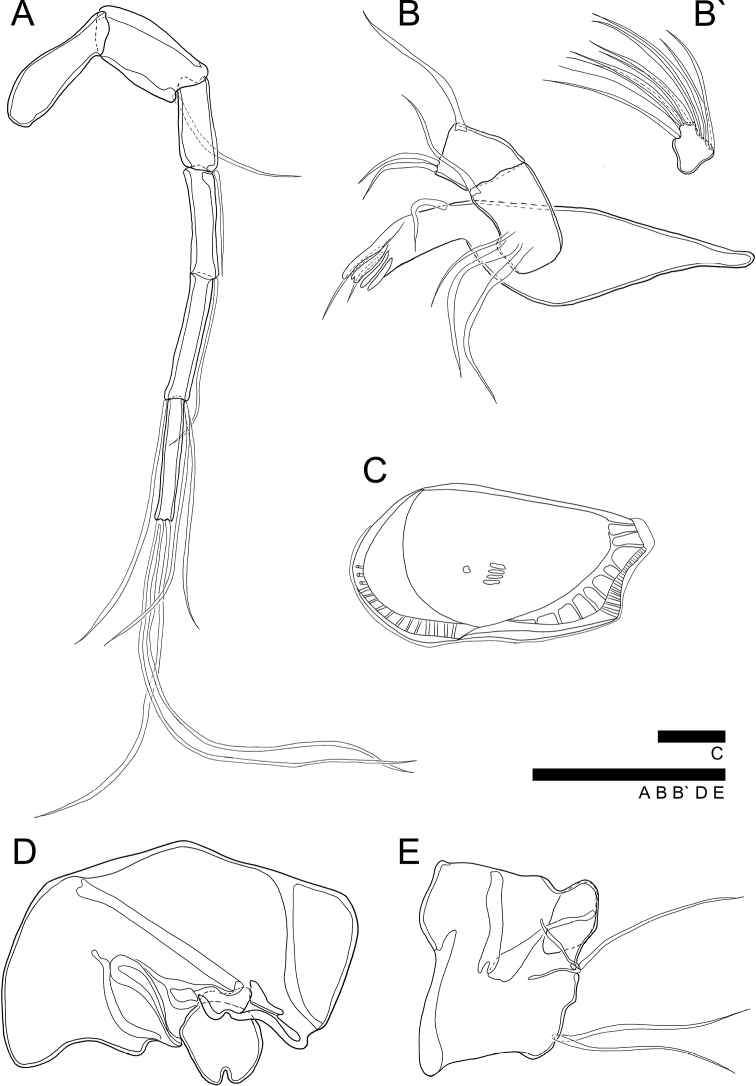
*Glacioloxoconchajisepoensis* sp. nov.: male (MABIKCR0025819) **A**A1**B**Md**B**' terminal segment of endopod **C**RV internal view **D**Hp, female (MABIKCR0025820) **E**GF. Scale bars: 50 µm (**A, B, B', D, E**); 100 µm (**C**).

**Figure 8.**
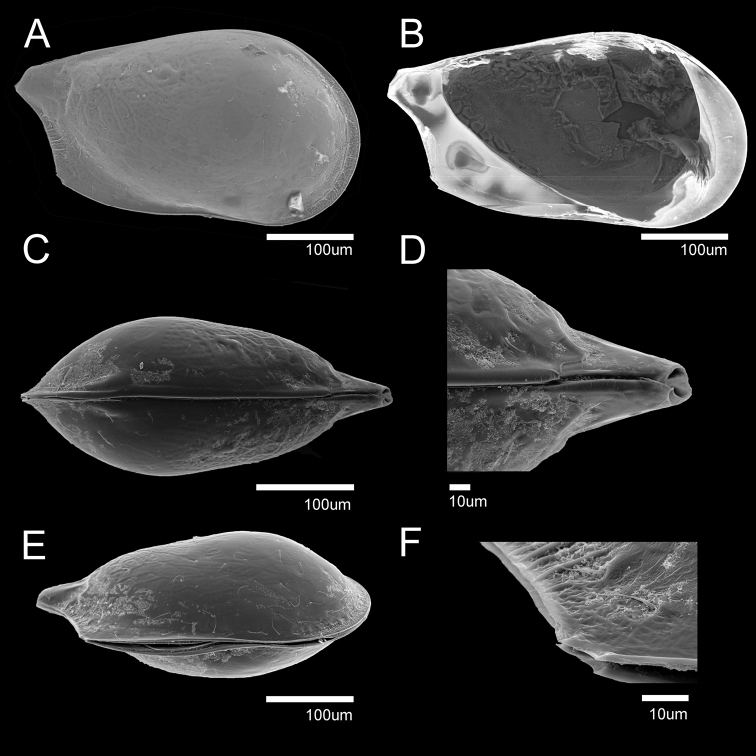
SEM photographs of *Glacioloxoconchajisepoensis* sp. nov.: male (paratype) **A**RV external view **B**LV internal view, male (paratype) **C** dorsal view **D** postero dorsal margin **E**RV internal view **F** posterior ventral margin.

**Male. *Carapace*** (Figs 7C, 8). Relatively small, L ~ 400 µm, H ~ 220 µm. RV overlapping LV dorsal margin (Fig. 8C). Carapace similar to that of *G.jeongokensis*. Some differences are that dorsal margin is slightly sloped (Fig. 8A, B), and the caudal process is slightly longer than that of *G.jeongokensis* (Fig. 8D). Anterior and posterior pore channel well developed (Fig. 7C). Muscular imprint same as *G.jeongokensis* (Figs 7C, 8B). Hinge adont (Fig. 7C).

***A1*** (Fig. 4A). Six-segmented. First segment without setulae or setae. Second segment with one bare seta postero-distally reaching 1/2 length of fourth segment. Third segment with one bare seta antero-distally reaching end of fourth segment. Fourth segment with one bare seta antero-distally reaching 1/3 length of terminal segment. Fifth segment with two bare setae on anterior-distal margin almost 2 × longer than terminal segment, one bare seta postero-distally almost 2 × longer than terminal segment. Terminal segment with three long bare setae on distal margin, almost 3.5 × longer than terminal segment. L ratios between six segments 1.4:1.2:1:1.2:1.3:1.3.

***Md*** (Fig. 7B, B'). Coxa slightly crushed shape with four strong teeth and two thin bare setae on distal margin and one bare seta near anterior distal margin. Exopod with three bare setae; endopod three-segmented. First segment with one bare seta antero-distally. Second segment with two bare setae antero-distally and one bare seta postero-distally. Terminal segment with ten setae, four of which arise from anterior margin, four from distal margin and two from postero-distal margin. First segment almost 2 × longer than terminal segment.

***Hp*** (Fig. 7D). Similar to *G.jeongokensis* but smaller. CR lost.

Other appendages same as in *G.jeongokensis* sp. nov.

A2 Four-segmented. L ratios between four segments 10: 3.5: 12.3: 1.

L5 Four-segmented. L ratios between four segments 3.6: 2: 1: 1.4.

L6 Four-segmented. L ratios between four segments 3.4: 2.2: 1: 1.4.

L7 Four-segmented. L ratios between four segments 3.6: 2.9: 1: 1.8.

**Female.** Carapace broken.

***GF*** (Fig. 5E). Basal part rectangular form. Three bare setae of CR observed. Ovary sub-rectangular.

All other appendages same as in male.

#### Genus *Loxocauda* Schornikov, 1969

##### ﻿ Loxocauda orientalis

Taxon classificationAnimaliaPodocopidaLoxoconchidae

Schornikov, 2011

AF49ED0B-A2DB-5826-92BA-BFF4085EB3AE

Figs 9 , 10

Loxocauda sp. – Schornikov 2006: 43; Zenina 2009: 307.
Loxocauda sp. 6 – Lee et al. 2000: 465.
Loxocauda sp. 9 – Lee et al. 2000: 466.
Loxocauda orientalis Schornikov, 2011: 100.

###### Material examined.

One male, dissected on one slide and shell on micropaleontological slide from South Korea, Gyeongsangnam-do, Geoje-si, Geoje-myeon, Beopdongeogu-ro, Beopdong harbor. 34°49.252'N, 128°31.227'E, 5 Apr 2021, leg. Changgyun Yu & Byung-jin Yoo; two females, dissected on one slide each, and one male dissected on one slide, all shells on separate micropaleontological slides from South Korea, Jeollabuk-do, Buan-gun, Byeonsan-myeon, Saemangeum-ro, Garyuk-do harbor. 35°43.603'N, 126°31.770'E, 30 Apr 2021, leg. Hyunsu Yoo & Byung-jin Yoo.

###### Redescription.

**Figure 9.**
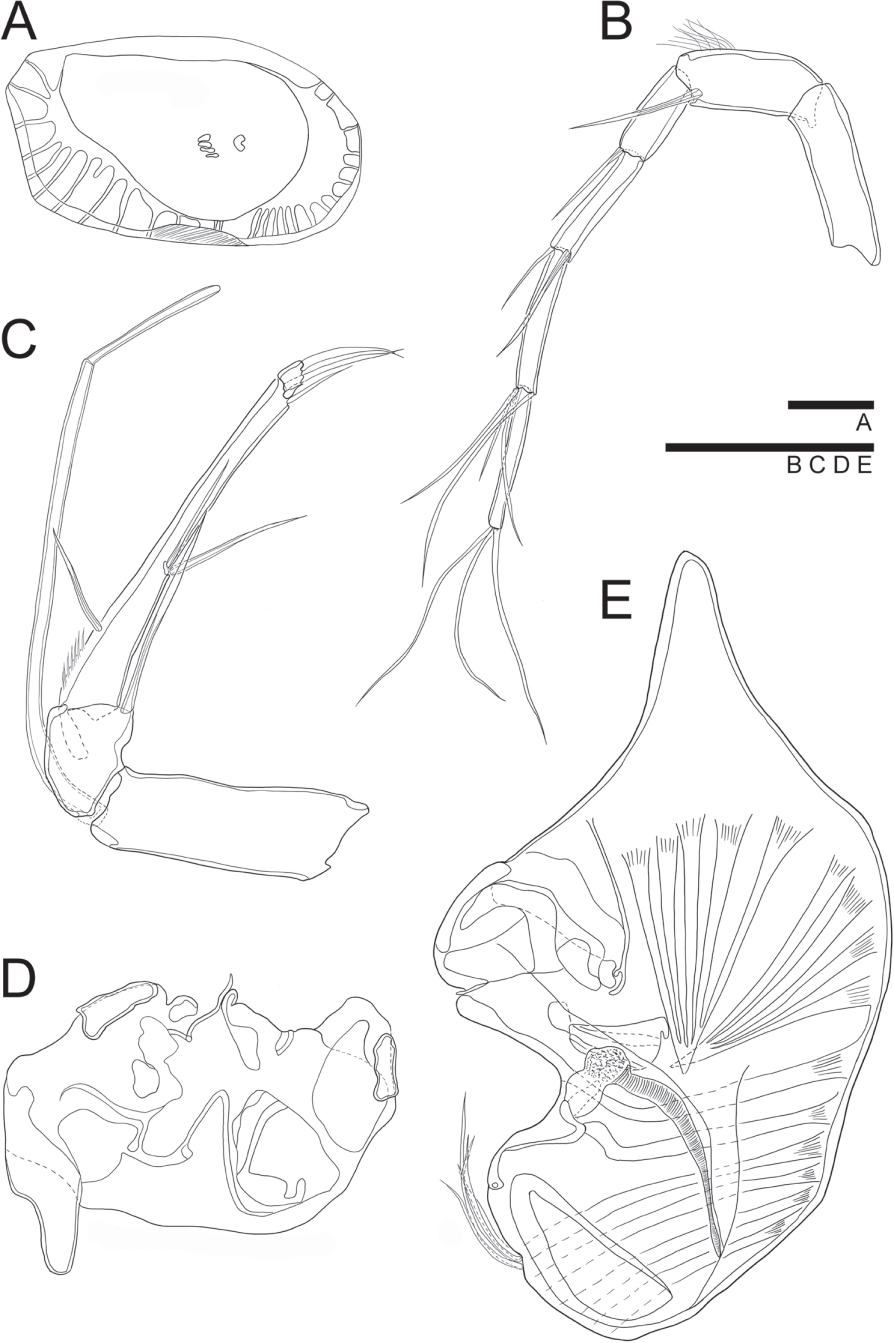
*Loxocaudaorientalis* Schornikov, 2011: male **A**RV internal view **B**A1**C**A2; female **D**GF; male **E**Hp. Scale bars: 50 µm (**B, C, D, E**); 100 µm (**A**).

**Figure 10.**
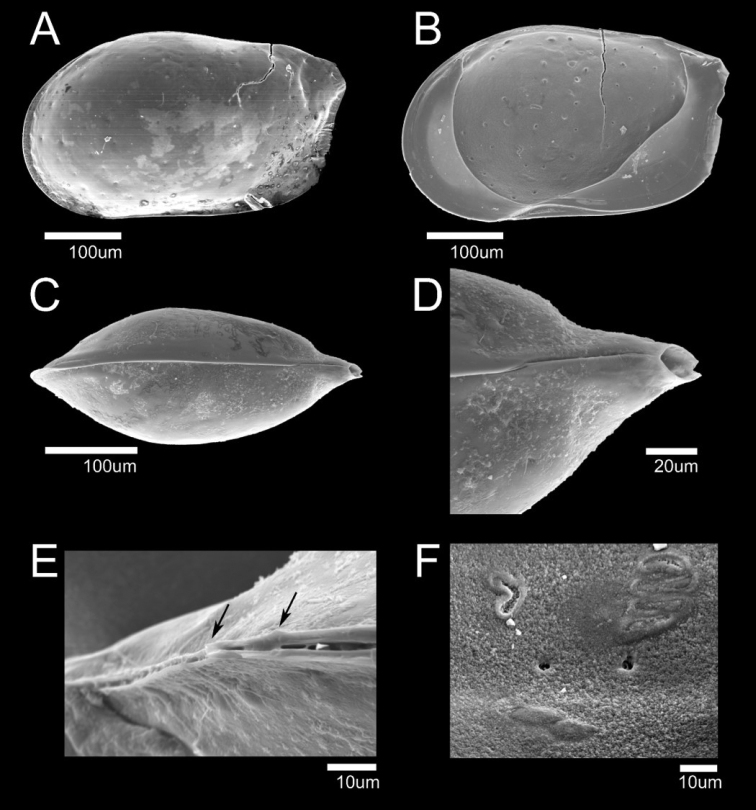
SEM photographs of *Loxocaudaorientalis* Schornikov, 2011: male **A**LV external view **B**RV internal view; male **C** dorsal view **D** posterior part of dorsal margin **E** postero-ventral margin (black arrow point to spines) **F** muscular scar print.

**Male. *Carapace*** (Figs 9A, 10). Larger than another *Glacioloxoconcha* species, L ~ 420 µm, H ~ 243 µm. RV overlapping LV dorsally. Carapace subquadrate in lateral view (Figs 9A, 10A, B). Anterior margin rounded, dorsal margin slightly arched and postero-dorsal margin with caudal process smaller than in *G.jeongokensis* sp. nov. (size almost 50 µm), ventral margin straight, postero-ventral margin with spine (Fig. 10E). Postero-ventral and anterior margins strongly compressed. Greatest H situated in front of middle. Surface smooth with few simple setae sporadically distributed (Fig. 10A). Marginal pore canals strongly developed and distributed from anterior to posterior margins (Fig. 9A). Fused zone situated medially on ventral margin, strongly developed. Muscular scar imprints consisting of a row of four vertical scars, one bent frontal scar, with two scars below it (Fig. 10F). Hinge adont (Fig. 9A).

***A1*** (Fig. 9B). Six-segmented. First segment without setulae and setae. Second segment with setulae on antero-distal and one bare seta on postero-distal margin, reaching 1/3 length of fourth segment. Third segment with one bare seta on antero-distally, reaching 3/4 length of fourth segment. Fourth segment with two bare setae, one antero-distally, reaching 1/2 length of next segment, another postero-distally, reaching 3/4 length of next segment. Penultimate segment with four bare setae, three setae antero-distally, and one on postero-distal margin, length of two setae on anteriorly reaching slightly over terminal segment, and one reaching 2/3 length of terminal segment, length of one seta on posteriorly over terminal segment. Terminal segment with three long, bare setae on distal margin, almost 1.5 × longer than terminal segment. L ratios between six segments 1.7: 1.3: 1: 1.2: 1.5: 1.6.

***A2*** (Fig. 9C). Four-segmented. Exopod transformed into two-segmented spinneret seta. First endopodal segment without setulae or seta. Second segment with one bare seta postero-distally reaching 2/3 of following segment. Third segment with setulae antero-proximally, and with one bare seta on antero-proximal margin, reaching 1/2 length of the same segment; two bare setae postero-medially, reaching 2/3 length of same segment, one bare seta postero-distally, almost 2.5 × longer than terminal segment, one bare seta on distal margin, almost 4 × longer than terminal segment. Terminal segment with one strong, bare claw on distal margin almost 4 × longer than same segment. L ratios between four segments: 7.1: 3: 11.4: 1.

***Hp*** (Fig. 9E). Basal plate subquadrate with flection antero-medially, four bare setae antero-distally, and strong muscle keeping tension between central and peripheral parts. Distal lobe sub-triangular. Ejaculatory process densely coiled.

**Female.** Larger than males. L ~ 484 µm, H ~ 287 µm. Shape and all other morphological features similar to male. Fused zone with three simple setulae.

***GF*** (Fig. 9D). Basal plate sub-rectangular and Ovary subquadrate. Without setulae and setae.

##### ﻿ Loxocauda cf. orientalis

Taxon classificationAnimaliaPodocopidaLoxoconchidae

55094FBD-A212-5F73-B258-5B10C46146A2

Figs 11 , 12

###### Material examined.

One male, dissected on one slide (NIBRIV0000882304), shell on micropaleontological slide (NIBRIV0000882314); one female, dissected on one slide (NIBRIV0000882310) and shell on micropaleontological slide (NIBRIV0000882312); one male and female dissected on slide each, and shells on micropaleontological slide each; South Korea, Gyeongsangnam-do, Geoje-si, Irun-myeon, Jisepohaean-ro, Jisepo harbor. 34°49.919'N, 128°42.220'E, 19 May 2020, leg. Hyunsu Yoo & Byung-jin Yoo.

###### Brief description.

**Figure 11.**
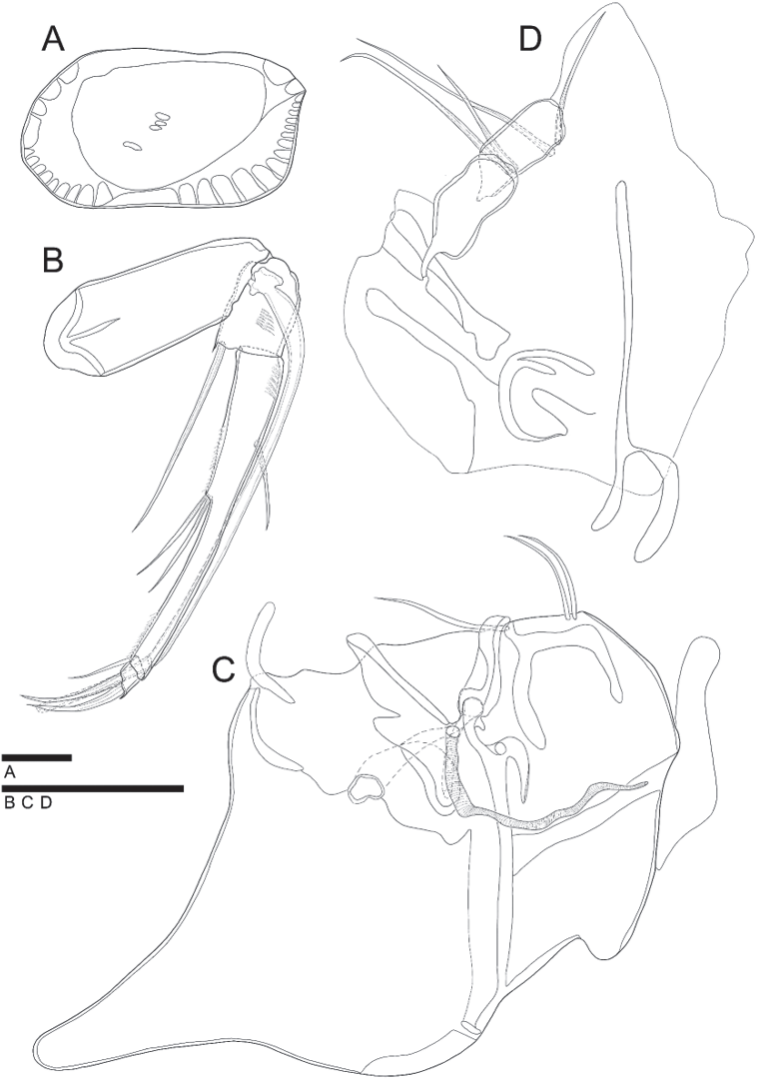
Loxocaudacf.orientalis: male (NIBRIV0000882304, NIBRIV0000882314) **A**RV internal view **B**A2**C**Hp; female (NIBR0000882310) **D**GF. Scale bars: 50 µm (**B, C, D**); 100 µm (**A**).

**Figure 12.**
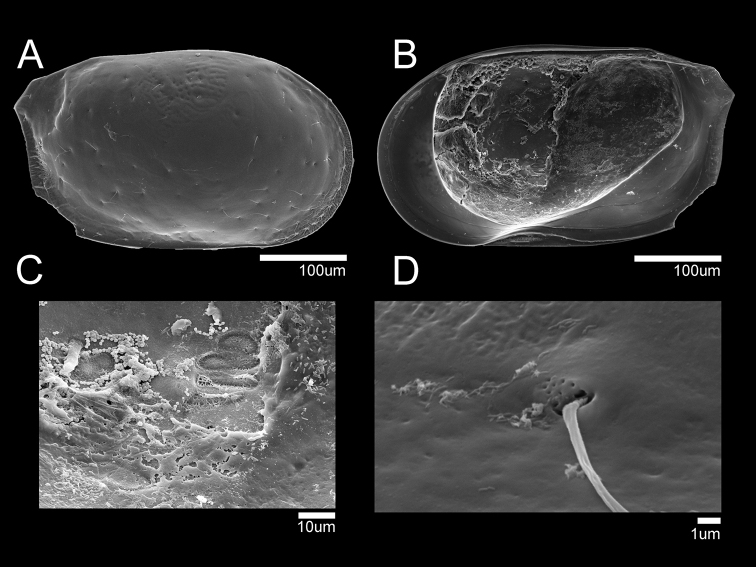
SEM photographs of Loxocaudacf.orientalis: male (NIBRIV0000882314) **A**RV external view **B**RV internal view **C** muscular scar print **D** simple seta pore on external carapace.

**Male.** Carapace same as in *L.orientalis* (Figs 11A, 12A, B). Differences include dorsal margin being straighter, and greatest H situated close medially. A2 (Fig. 11B) with well-developed setulae on second and third segments. Hp (Fig. 11C) with basal plate sub-rectangular and with three bare setae antero-distally. Lobe with sub-triangular form. No strong muscles present.

Other appendages same as in *L.orientalis* Schornikov, 2011

L ratios between segments as indicated below:

A1 Six-segmented. 2.4: 1.2: 1: 1.1: 1.4: 1.3.

A2 Four-segmented. 6.4: 2.9: 9.8: 1.

L5 Four-segmented. 3.5: 1.9: 1: 1.2.

L6 Four-segmented. 3.3: 2.4: 1: 1.3.

L7 Four-segmented. 2.4: 1.9: 1: 1.2.

**Figure 13.**
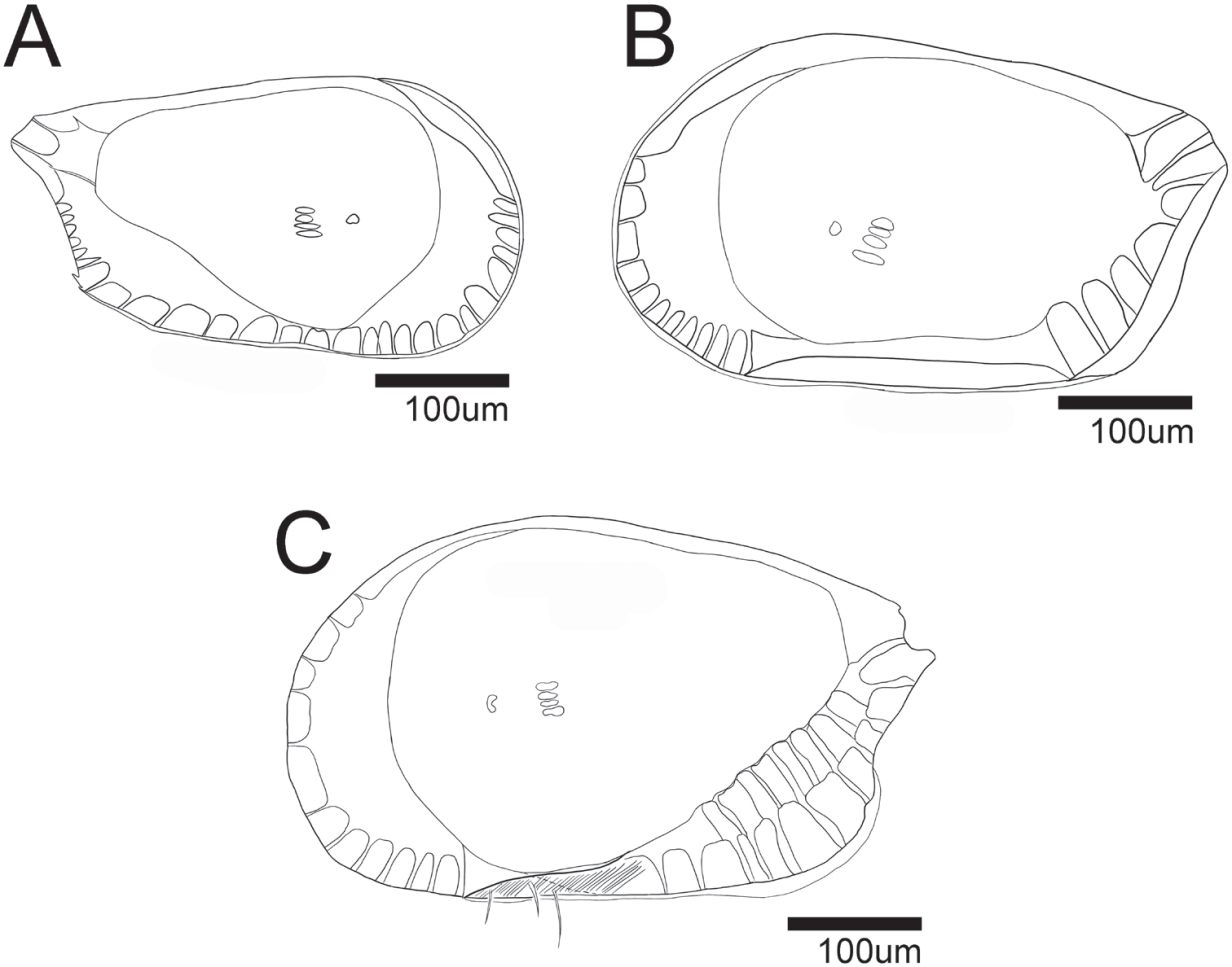
Internal view of female carapace **A***Glacioloxoconchajeongokensis* sp. nov. (NIBRIV0000882311 allotype) **B**Loxocaudacf.orientalis (NIBRIV0000882312) **C***Loxocaudaorientalis* Schornikov, 2011 (MABIKCR0025820).

**Female.** Larger than male (Fig. 13B). L ~ 459 µm, H ~ 267 µm. Shape and all other morphological features similar to male. GF illustrated in Fig. 11D. Basal plate sub-triangular. Ovary subquadrate. With three bare setae on antero-medial margin. All other appendages same as in male.

### ﻿Molecular analysis

Intraspecific pairwise distances (p-distances) of the COI sequences between specimens of *Glacioloxoconchajeongokensis*, *G.jisepoensis*, and *Loxocaudaorientalis* varied between 0 and 0.6% (Suppl. material 3). Interspecific p-distances between two new *Glacioloxoconcha* species were ~ 11%. Distances between COI sequences belonging to *Loxocaudaorientalis* and to *Glacioloxoconcha* varied between 21.0% and 24.1%. The COI alignment was 707 base pairs long and TVM+F+I+G4 model (Kalyaanamoorthy et al. 2017) was chosen as the best fit. The number of constant sites and parsimony informative sites were 296 and 375, respectively.

**Figure 14.**
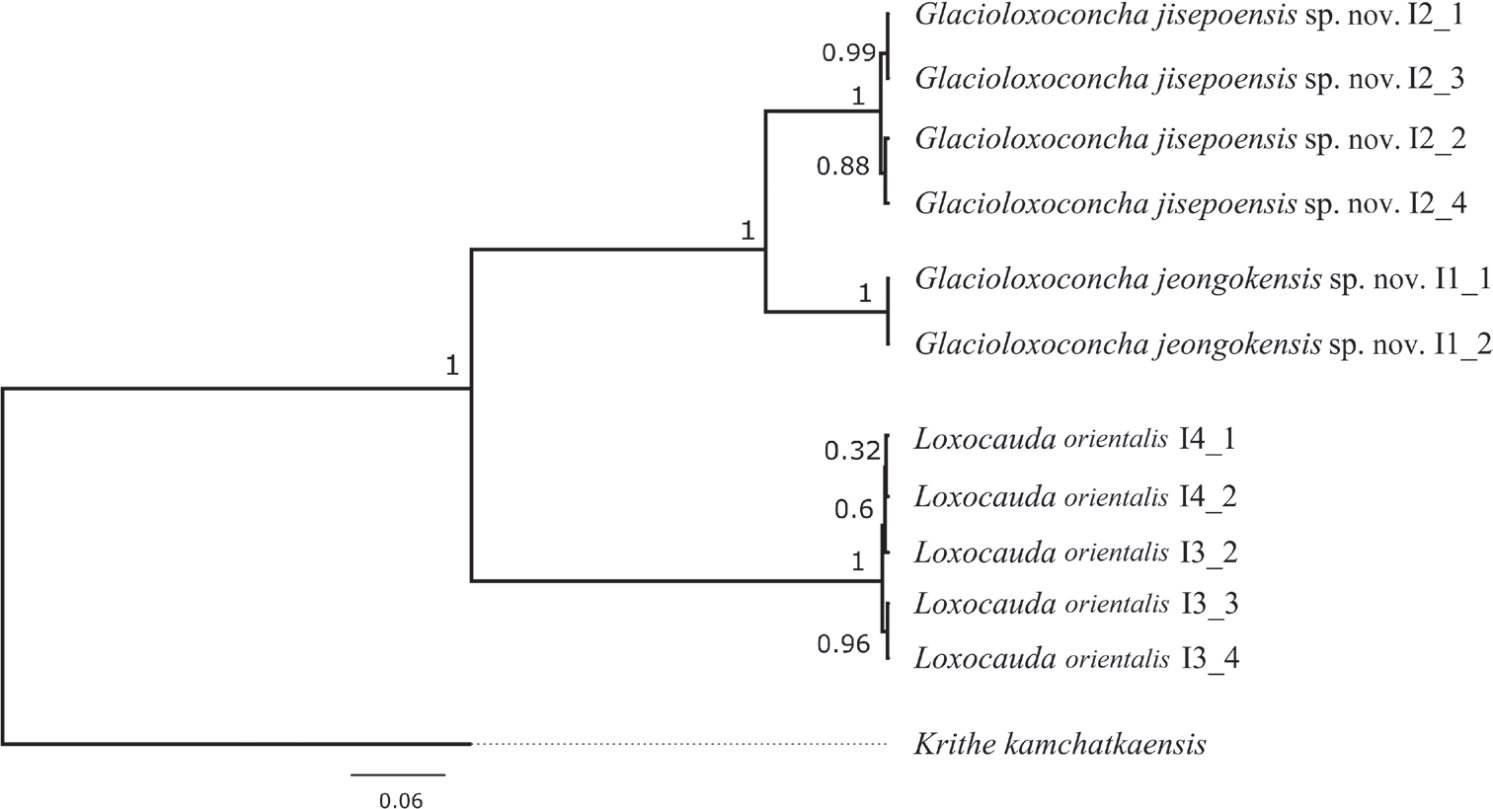
Bayesian inference rooted cladogram of three Loxocaudinae species constructed from the COI dataset. Numbers above branches represent posterior probabilities.

*Glacioloxoconcha* and *Loxacauda* clustered separately on the tree (Fig. 14) and their respective branches received the maximum support. Similarly, two new *Glacioloxoconcha* species formed two well-supported clades.

The p-distances between *Loxocauda* and *Glacioloxoconcha* 18S rRNA sequences (Suppl. material 4) were much smaller and varied from 0.3% to 0.5%. Some differences (between 0.1% and 0.3%) were also recorded between two *Glacioloxoconcha* species, as well as between specimens belonging to the same species of this genus.

The final 18S alignment was 1972 base pairs long and it included the two new *Glacioloxoconcha* species and *L.orientalis*, in addition to 46 Cytherocopina taxa and an outgroup. The substitution model, TIM2+F+I+G4 (Kalyaanamoorthy et al. 2017) with gamma distribution was identified as the best fit for the evolutionary model. The number of constant sites was 1235 and the number of parsimony informative sites was 517.

**Figure 15.**
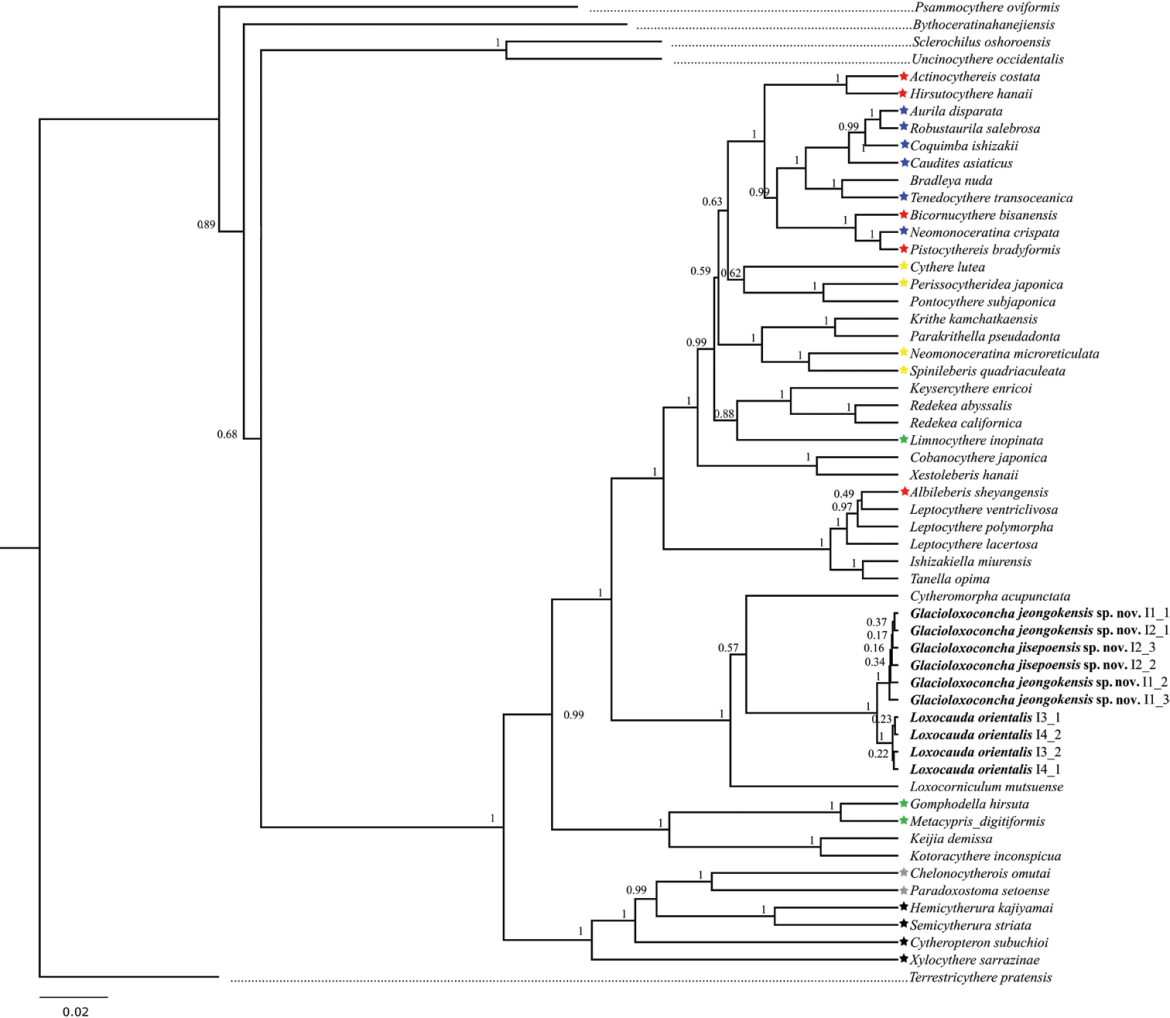
Bayesian inference rooted cladogram of the superfamily Cytheroidea constructed from the 18S rRNA dataset. Numbers above branches represent posterior probability. Stars indicate representatives of polyphyletic/paraphyletic families: red, Trachyleberididae; blue, Hemicytheridae, yellow, Cytheridae; green, Limnocytheridae; grey, Paradoxoxtomatidae; black, Cytheruridae.

The phylogenetic tree based on the 18S alignment (Fig. 15), strongly supports the monophyly of the family Loxoconchidae, which for this analysis only included one species each of *Cytheromorpha* Hirchmann, 1909, *Loxocorniculum* Benson & Coleman, 1963, and *Loxocauda*, as well as two new *Glacioloxoconcha* species. Our choice of the outgroup did not strongly support the monophyly of the ingroup taxa (Cytheroidea), as this branch had a posterior probability of only 0.89. The tree also indicates a polyphyletic and paraphyletic nature of several families (see discussion).

## ﻿Discussion

In contrary to *Glacioloxoconchasuedshetlandensis*, the two new *Glacioloxoconcha* species from Korea have a distinct tail-like extension on each valve. Other differences include a more robust A2 in the new species, and the presence of three rays on the exopodite of the Md vs. one ray in *G.suedshetlandensis* (see Hartmann 1990; Schornikov 2011b). The chaetotaxy and the morphology of the Md exopodite has been widely used to distinguish cytheroid taxa on various systematic levels (Karanovic and Brandão 2015). Considering how delicate this part is and how easily it is lost or folded during dissection, it should not be the primary character used in cytheroids classification. All three *Glacioloxoconcha* species have a very similar hemipenis, which is the reason for placing the Korean species into a genus that has been known only from Antarctica. Such a wide distribution, especially of the cytheroid ostracods, seems to be relatively common (Brandão and Päplow 2011; Brandão and Karanovic 2015). For example, Yamaguchi (2000) examined the wide distribution of the genus *Ishizakiella* McKenzie & Sudijono, 1981, which has one species living in New Zealand and three in Japan and Korea (see Yoo et al. 2012), and suggested that the ancestor of the Japanese species colonized the islands before the Pleistocene glaciation, and subsequently diverged there. Tanaka et al. (2018) showed that some ostracod species endemic to Japan were transported across the Pacific on the tsunami debris from the 2011 earthquake. On the other hand, a wide geographic distribution of some taxa (especially species) should be taken with caution. Namely, recent studies showed that after the reexamination of the type material and various world records, a wide distribution is questionable (Brandão and Yasuhara 2013). The two new species are very similar, but *G.jeongokensis* has a slightly longer tail-like extension on the shell, a different chaetotaxy on the A1, and a larger hemipenis with a sinusoid ventral margin of the distal lobe. The two species also have high COI distances of ~ 11%, which has been suggested as good evidence for species delineation (da Silva et al. 2011; Léfebure et al. 2016). *Glacioloxoconcha* species are very closely related and seems to have allopatric distributions, which is supported by small morphological differences in all soft body parts, including the hemipenis. The character displacement phenomenon, where differences in sexual characters are enlarged in the case of overlapping distribution, was already noticed for the cytheroid ostracods (Tsukagoshi 1988).

*Loxocaudaorientalis* and L.cf.orientalis are very similar both in the carapace and the soft body parts morphologies and differ only in few details. Loxocaudacf.orientalis does not have strong muscles on the hemipenis and has a slightly different shape of the female CR and genital lobe. Study of the ontogeny of another Loxoconchidae member, *Loxoconchajaponica* Ishizaki, 1968, does not mention any changes in the development of the hemipenis musculature after the last molt. But, as Yamada et al. (2014) showed, several morphological changes do occur in the development of the Zenker organ of a Candonidae ostracod after reaching the adult stage. The function of the Zenker organ in Cypridoidea is to pump the sperm, a role that is in Cytheroidea taken up by the hemipenis (see Meisch 2000). There are also some subtle differences in the dorsal margins of the male valves: in the L.cf.orientalis this margin is slightly flatter than in *L.orientalis.* Unfortunately, COI could not be successfully amplified for L.cf.orientalis to support our decision, and more material is needed.

The similarities and character overlap between *Glacioloxoconcha*, *Loxocauda*, and *Loxoconcha* Sars, 1926 question not only the validity of the subfamily Loxocaudinae within Loxoconchidae, but also the validity of *Glacioloxoconcha* and *Loxocauda*. On our reconstructed phylogenetic tree of Cytheroidea, the two genera are separated, but together form a well-supported clade. In addition, their respective branches are very short, especially in comparison to other two Loxoconchidae genera included in the analysis. One of them, *Loxocorniculum* Benson & Coleman, 1963 is currently accepted as a subgenus of *Loxoconcha* (see Bate et al. 1981). The phylogenetic tree does not include two 18S sequences attributed to *Loxoconcha* sp. (GenBank accession numbers AY191447 and AY455769) because of the incomplete identification, and also because our attempt to include them resulted in *Loxoconcha* clustering with sequences belonging to *Aurila* sp., *Pistocythereis* sp., *Limnocythere* sp.1, *Neomonoceratina* sp., and *Bicornucythere* sp. This indicates a potential misidentification not only of *Loxoconcha* but other unrelated genera.

Polyphyletic and/or paraphyletic nature of several families on the phylogenetic tree can partially be explained by misidentification. For example, *Albileberissheyangensis* Chen in Hou, Chen, Yang, Ho, Zhou & Tian, 1982, a species belonging to the family Trachyleberididae, clusters within the family Leptocytheridae (Fig. 15). The two families have many prominent morphological differences in both the soft part and the shell morphologies and the position of a Trachyleberididae species within Leptocytheridae can only be a result of misidentification. On the other hand, polyphyly of the families Cytheridae, Hemicytheridae, Trachyleberididae, and Limnocytheridae on the tree (see different star colors on the tree, Fig. 15) is the result of unstable systematics and indicates the necessity for revisions. Trachyleberididae is a very diverse Mesozoic taxon, and a recent revision of the *Trachyleberis* Brady, 1898 type species (Brandão et al. 2013) contributed to a better understanding not only of this genus’ systematics, but also of the family. Tanaka et al. (2021) studied a deep-sea member of the family Keysercytheridae, and their phylogenetic reconstruction showed that Limnocytheridae as well as Paradoxostomatidae are polyphyletic and proposed some systematic changes for the latter family. On our tree Paradoxostomatidae clusters with Cytheruridae (grey star on the tree, Fig. 15). However, *Xylocytheresarrazinae* Tanaka, Lelièvre & Yasuhara, 2019, a member of the family Cytheruridae, is a sister taxon to both Cytheruridae and Paradoxostomatidae, rendering the former family paraphyletic.

From both our and previous studies it is clear that several families belonging to Cytheroidea need a revision, which should combine morphology of both shell and soft parts (Karanovic and Brandão 2015). However, such revision is difficult as most cytheroids ostracods are known from the fossil record only and this would only partially resolve the problems. Nevertheless, Recent taxa could provide an insight into the morphological evolution of Cytheroidea and offer some solution, especially if geometric morphometrics of the shell are used as an aid in this revision.

## Supplementary Material

XML Treatment for Glacioloxoconcha jeongokensis

XML Treatment for Glacioloxoconcha jisepoensis

XML Treatment for Loxocauda orientalis

XML Treatment for Loxocauda cf. orientalis

## Supplementary materials

Supplementary material 1 List of primers and PCR conditions Data type table (word document) https://binary.pensoft.net/file/791650This dataset is made available under the Open Database License (http://opendatacommons.org/licenses/odbl/1.0/). The Open Database License (ODbL) is a license agreement intended to allow users to freely share, modify, and use this Dataset while maintaining this same freedom for others, provided that the original source and author(s) are credited.Hyunsu Yoo, Pham Thi Minh Huyen, Jinho Chae, Ivana Karanovic

Supplementary material 2 List of 18S and COI sequences used for phylogenetic analysis Data type table (word document) https://binary.pensoft.net/file/791651This dataset is made available under the Open Database License (http://opendatacommons.org/licenses/odbl/1.0/). The Open Database License (ODbL) is a license agreement intended to allow users to freely share, modify, and use this Dataset while maintaining this same freedom for others, provided that the original source and author(s) are credited.Hyunsu Yoo, Pham Thi Minh Huyen, Jinho Chae, Ivana Karanovic

Supplementary material 3 Pairwise p-distances among COI sequences of three new Loxocaudinae species Data type table (word document) https://binary.pensoft.net/file/791652This dataset is made available under the Open Database License (http://opendatacommons.org/licenses/odbl/1.0/). The Open Database License (ODbL) is a license agreement intended to allow users to freely share, modify, and use this Dataset while maintaining this same freedom for others, provided that the original source and author(s) are credited.Hyunsu Yoo, Pham Thi Minh Huyen, Jinho Chae, Ivana Karanovic

Supplementary material 4 Pairwise p-distances among 18S sequences of three new Loxocaudinae species Data type table (word document) https://binary.pensoft.net/file/791653This dataset is made available under the Open Database License (http://opendatacommons.org/licenses/odbl/1.0/). The Open Database License (ODbL) is a license agreement intended to allow users to freely share, modify, and use this Dataset while maintaining this same freedom for others, provided that the original source and author(s) are credited.Hyunsu Yoo, Pham Thi Minh Huyen, Jinho Chae, Ivana Karanovic
