# Supplementary material for: ﻿Three Loxocaudinae species (Ostracoda, Podocopida) from South Korea
Source: Zookeys. 2023 Jan 6;1138:183–209. doi: 10.3897/zookeys.1138.96201 (PMC9840065; doi:10.3897/zookeys.1138.96201)
Supplement: Supplementary material 1 — List of primers and PCR conditions [file zookeys-1138-183_article-96201__-s001.docx]

**Supplementary file 1.** List of primers and PCR conditions.

| Markers | | Name of primer | Sequence (5'-3') | Taq polymerase | PCR amplification conditions | Reference |
| --- | --- | --- | --- | --- | --- | --- |
| Mitochonrial markers | COI | COI_PsF | AAA TCA TAA AGA TAT TGG CAC | HotStar Taq (Qiagen) | 95°C-15 min; [94°C -30s; 46°C-45s; 72°C-1 min] 40 cycles; 72°C- 10 min; 4°C- ∞ | This study |
|  |  | COI_PsR | ATT ACG ATC TGT TAA AAG TAT TGT G |  |  |  |
|  |  | LCO1490 | GGT CAA CAA ATC ATA AAG ATA TTG G |  |  | (Folmer et al. 1994) |
|  |  | HCO2198 | TAA ACT TCA GGG TGA CCA AAA AAT CA |  |  |  |
| Nuclear markers | 18S | P1 | CCT GGT TGA TCC TGC CAG | PCR Premix (Bioneer Inc.) | 95°C-5 min; [94°C -30s; 48°C-45s; 72°C-1 min] 40 cycles; 72°C- 5 min; 4°C- ∞ | (Yu et al. 2006) |
|  |  | P1W1 | ATA CGG GAC TCA TCC GAG |  |  |  |
|  |  | P2W1 | GCT ACA GTC TCG TTC GTT ATC |  |  |  |
|  |  | P2 | TAA TGA TCC TTC CGC AGG TT |  |  |  |
