# Supplementary material for: ﻿Three Loxocaudinae species (Ostracoda, Podocopida) from South Korea
Source: Zookeys. 2023 Jan 6;1138:183–209. doi: 10.3897/zookeys.1138.96201 (PMC9840065; doi:10.3897/zookeys.1138.96201)
Supplement: Supplementary material 2 — List of 18S and COI sequences used for phylogenetic analysis [file zookeys-1138-183_article-96201__-s002.docx]

**Supplementary file 2.** List of 18S and COI sequences used for phylogenetic analysis.

| Species | Superfamily; Family | GenBank numbers | | GPS coordinates | Country | Source |
| --- | --- | --- | --- | --- | --- | --- |
|  |  | COI | 18S |  |  |  |
| *Actinocythereis costata* | Cytheroidea; Trachyleberididae |  | AB076652 | 15°14.8′N 145°44.0′E | Japan | Yamaguchi, 2003 |
| *Albileberis sheyangensis* | Cytheroidea; Cytherideidae |  | AY863436 | - | China | Yu et al. 2006 |
| *Aurila disparata* | Cytheroidea; Hemicytheridae |  | AB076643 | 35°09.3′N 139°36.9′E | Japan | Yamaguchi, 2003 |
| *Bicornucythere bisanensis* | Cytheroidea; Trachyleberididae |  | AB076649 | 35°08.9′N 139°36.6′E | Japan | Yamaguchi, 2003 |
| *Bradleya nuda* | Cytheroidea; Thaerocytheridae |  | AB076647 | 33°37.1′N 136°01.5′E | Japan | Yamaguchi, 2003 |
| *Bradleycypris vittata* | Cypridoidea; Cyprididae |  | KM403072 | 37°25′5′′N 127°3′55.2′′E | South Korea | Kong et al. 2014 |
| *Bythoceratina hanejiensis* | Cytheroidea; Bythocytheridae |  | AB076619 | 33°42.2′N 135°16.6′E | Japan | Yamaguchi, 2003 |
| *Caudites asiaticus* | Cytheroidea; Hemicytheridae |  | AB076646 | 26°35.2′N 128°09.6′E | Japan | Yamaguchi, 2003 |
| *Chelonocytherois omutai* | Cytheroidea; Paradoxostomatidae |  | LC380021 | 30°24'56.2"N 130°26'21.8"E | Japan | Tanaka and Hayashi, 2019 |
| *Cobanocythere japonica* | Cytheroidea; Xestoleberididae |  | AB076634 | 33°41.3′N 135°20.4′E | Japan | Yamaguchi, 2003 |
| *Coquimba ishizakii* | Cytheroidea; Hemicytheridae |  | AB076645 | - | - | Yamaguchi, 2003 |
| *Cythere lutea* | Cytheroidea; Cytheridae |  | AB076636 | 51°23.4′N 1°25.3′W | England | Yamaguchi, 2003 |
| *Cytheromorpha acupunctata* | Cytheroidea; Loxoconchidae |  | AB076630 | 37°18′N 137°14′E | Japan | Yamaguchi, 2003 |
| *Cytheropteron subuchioi* | Cytheroidea; Cytheruridae |  | AB076628 | 35°08.3′N 139°34.9′E | Japan | Yamaguchi, 2003 |
| ***Glacioloxoconcha jeongokensis* sp. nov.** | **Cytheroidea; Loxoconchidae** | **ON103621; ON103622** | **ON113965; ON113966; ON113967** | **37°11'10.7"N 126°39'01.4"E** | **South Korea** | **This study** |
| ***Glacioloxoconcha jisepoensis* sp. nov.** | **Cytheroidea; Loxoconchidae** | **ON103623; ON103624; ON103625; ON103626** | **ON113968; ON113969; ON113970** | **34°49'55.16"N 128°42'13.20"E** | **South Korea** | **This study** |
| *Gomphodella hirsuta* | Cytheroidea; Limnocytheridae |  | MW338924 | 34°20'53"S 115°09'14"E | Australia | Karanovic, 2006 |
| *Hemicytherura kajiyamai* | Cytheroidea; Cytheruridae |  | AB076627 | 35°09.3′N 139°36.9′E | Japan | Yamaguchi, 2003 |
| *Hirsutocythere hanaii* | Cytheroidea; Trachyleberididae |  | AB076653 | - | Japan | Yamaguchi, 2003 |
| *Ishizakiella miurensis* | Cytheroidea; Leptocytheridae |  | AB076632 | 38°11.3′N 140°56.3′E | Japan | Yamaguchi, 2003 |
| *Keijia demissa* | Cytheroidea; Eucytheridae |  | AB076622 | 26°35.2′N 128°09.6′E | Japan | Yamaguchi, 2003 |
| *Keysercythere enricoi* | Cytheroidea; Keysercytheridae |  | MW338924 | 43°48.43′N 151°44.35′E to 43°47.64′N 151°44.51′E | Japan | Tanaka et al. 2021 |
| *Kotoracythere inconspicua* | Cytheroidea; Eucytheridae |  | AB076621 | 26°35.2′N 128°09.6′E | Japan | Yamaguchi, 2003 |
| *Krithe kamchatkaensis* | Cytheroidea; Krithidae | MK443402 | MK584872 |  | Japan | Yoo et al. 2019 |
| *Leptocythere lacertosa* | Cytheroidea; Leptocytheridae |  | AB076631 | 51°19.1′N 1°22.7′W | Japan | Yamaguchi, 2003 |
| *Leptocythere polymorpha* | Cytheroidea; Leptocytheridae |  | AB674963 | - | Japan | Hiruta et al. 2016 |
| *Leptocythere ventriclivosa* | Cytheroidea; Leptocytheridae |  | AY863435 | - | China | Yu et al. 2006 |
| *Limnocythere inopinata* | Cytheroidea; Limnocytheridae |  | KX228783 | - | - | Unpublished |
| ***Loxocauda orientalis*** | **Cytheroidea; Loxoconchidae** | **ON103627; ON103628; ON103629; ON103630; ON103631** | **ON113971; ON113972; ON113973; ON113974** | **34°49'16.91"N 128°31'13.62"E; 35°43'36.20"N 126°31'46.18"E** | **South Korea** | **This study** |
| *Loxocorniculum mutsuense* | Cytheroidea; Loxoconchidae |  | AB076629 | 33°41.3′N 135°20.3′E | Japan | Yamaguchi, 2003 |
| *Metacypris digitiformis* | Cytheroidea; Limnocytheridae |  | AB674964 | 43°06′17″N 144°20′11″E | Japan | Hiruta et al. 2016 |
| *Neomonoceratina crispata* | Cytheroidea; Cytheridae |  | DQ531763 | - | China | Unpublished |
| *Neomonoceratina microreticulata* | Cytheroidea; Cytheridae |  | AB076637 | 26°36.8′N 128°01.3′E | Japan | Yamaguchi, 2003 |
| *Paradoxostoma setoense* | Cytheroidea; Paradoxostomatidae |  | AB076623 | 35°08.9′N 139°36.6′E | Japan | Yamaguchi, 2003 |
| *Parakrithella pseudadonta* | Cytheroidea; Krithidae |  | AB076639 | 35°08.9′N 139°36.6′E | Japan | Yamaguchi, 2003 |
| *Perissocytheridea japonica* | Cytheroidea; Cytherideidae |  | AB076642 | 38°11.3′N 140°56.3′E | Japan | Yamaguchi, 2003 |
| *Pistocythereis bradyformis* | Cytheroidea; Trachyleberididae |  | AB076650 | - | - | Yamaguchi, 2003 |
| *Pontocythere subjaponica* | Cytheroidea; Cushmanideidae |  | AB076640 | 33°40.7′N 135°19.7′E | Japan | Yamaguchi, 2003 |
| *Psammocythere oviformis* | Cytheroidea; Psammocytheridae |  | AB674961 | - | Japan | Hiruta et al. 2016 |
| *Redekea abyssalis* | Cytheroidea; Paradoxostomatidae |  | MW338929 | 43°48.43′N 151°44.35′E to 43°47.64′N 151°44.51′E | Japan | Tanaka et al. 2021 |
| *Redekea californica* | Cytheroidea; Paradoxostomatidae |  | MW338929 | - | Japan | Tanaka et al. 2021 |
| *Robustaurila salebrosa* | Cytheroidea; Hemicytheridae |  | AB076644 | - | - | Yamaguchi, 2003 |
| *Schlerochilus oshoroensis* | Cytheroidea; Bythocytheridae |  | AB076620 | 43°12.2′N 140°52.6′E | Japan | Yamaguchi, 2003 |
| *Semicytherura striata* | Cytheroidea; Cytheruridae |  | AB076625 | - | Japan | Yamaguchi, 2003 |
| *Spinileberis quadriaculeata* | Cytheroidea; Cytheridae |  | AB076638 | 26°36.8′N 128°01.3′E | Japan | Yamaguchi, 2003 |
| *Tanella opima* | Cytheroidea; Leptocytheridae |  | AY863434 | - | China | Yu et al. 2006 |
| *Terrestricythere pratensis* | Terrestricytheroidea; Terrestricytheridae |  | AB674959 | 43°28′32″N 145°14′22″E | Japan | Hiruta et al. 2016 |
| *Tenedocythere transoceanica* | Cytheroidea; Thaerocytheridae |  | AB076648 | 26°35.2′N 128°09.6′E | Japan | Yamaguchi, 2003 |
| *Uncinocythere occidentalis* | Cytheroidea; Entocytheridae |  | AB674962 | - | Japan | Hiruta et al. 2016 |
|  |  |  |  |  |  |  |
| *Xestoleberis hanaii* | Cytheroidea; Xestoleberididae |  | AB076633 | 35°09.3′N 139°36.9′E | Japan | Yamaguchi, 2003 |
| *Xylocythere sarrazinae* | Cytheroidea; Cytheruridae |  | LC380020 | 47°56'57.5"N 129°05'54.4"E | Japan | Tanaka et al. 2019 |
