# Supplementary material for: ﻿Three Loxocaudinae species (Ostracoda, Podocopida) from South Korea
Source: Zookeys. 2023 Jan 6;1138:183–209. doi: 10.3897/zookeys.1138.96201 (PMC9840065; doi:10.3897/zookeys.1138.96201)
Supplement: Supplementary material 3 — Pairwise p-distances among COI sequences of three new Loxocaudinae species [file zookeys-1138-183_article-96201__-s003.docx]

**Supplementary file 3.** Pairwise p-distances among COI sequences of three new Loxocaudinae species.

| *Glacioloxoconcha jeongokensis*_I11 |  |  |  |  |  |  |  |  |  |  |
| --- | --- | --- | --- | --- | --- | --- | --- | --- | --- | --- |
| *Glacioloxoconcha jeongokensis*_I12 | 0.000 |  |  |  |  |  |  |  |  |  |
| *Glacioloxoconcha jisepoensis*_I21 | 0.119 | 0.119 |  |  |  |  |  |  |  |  |
| *Glacioloxoconcha jisepoensis*_I23 | 0.119 | 0.119 | 0.000 |  |  |  |  |  |  |  |
| *Glacioloxoconcha jisepoensis*_I22 | 0.117 | 0.117 | 0.002 | 0.002 |  |  |  |  |  |  |
| *Glacioloxoconcha jisepoensis*_I24 | 0.117 | 0.117 | 0.006 | 0.006 | 0.004 |  |  |  |  |  |
| *Loxocauda orientalis*_I32 | 0.239 | 0.239 | 0.214 | 0.214 | 0.214 | 0.210 |  |  |  |  |
| *Loxocauda orientalis*_I33 | 0.241 | 0.241 | 0.216 | 0.216 | 0.216 | 0.212 | 0.002 |  |  |  |
| *Loxocauda orientalis*_I34 | 0.241 | 0.241 | 0.216 | 0.216 | 0.216 | 0.212 | 0.002 | 0.000 |  |  |
| *Loxocauda orientalis*_I41 | 0.239 | 0.239 | 0.214 | 0.214 | 0.214 | 0.210 | 0.000 | 0.002 | 0.002 |  |
| *Loxocauda orientalis*_I42 | 0.239 | 0.239 | 0.214 | 0.214 | 0.214 | 0.210 | 0.000 | 0.002 | 0.002 | 0.000 |
