# Supplementary material for: ﻿Three Loxocaudinae species (Ostracoda, Podocopida) from South Korea
Source: Zookeys. 2023 Jan 6;1138:183–209. doi: 10.3897/zookeys.1138.96201 (PMC9840065; doi:10.3897/zookeys.1138.96201)
Supplement: Supplementary material 4 — Pairwise p-distances among 18S sequences of three new Loxocaudinae species [file zookeys-1138-183_article-96201__-s004.docx]

**Supplementary file 4.** Pairwise p-distances among 18S sequences of three new Loxocaudinae species.

| *Glacioloxoconcha jeongokensis*_I11 |  |  |  |  |  |  |  |  |  |
| --- | --- | --- | --- | --- | --- | --- | --- | --- | --- |
| *Glacioloxoconcha jeongokensis*_I12 | 0.000 |  |  |  |  |  |  |  |  |
| *Glacioloxoconcha jeongokensis*_I13 | 0.002 | 0.002 |  |  |  |  |  |  |  |
| *Glacioloxoconcha jisepoensis*_I21 | 0.002 | 0.002 | 0.003 |  |  |  |  |  |  |
| *Glacioloxoconcha jisepoensis*_I22 | 0.000 | 0.000 | 0.002 | 0.002 |  |  |  |  |  |
| *Glacioloxoconcha jisepoensis*_I23 | 0.001 | 0.001 | 0.003 | 0.003 | 0.001 |  |  |  |  |
| *Loxocauda orientalis*_I31 | 0.003 | 0.003 | 0.005 | 0.005 | 0.003 | 0.004 |  |  |  |
| *Loxocauda orientalis*_I32 | 0.003 | 0.003 | 0.005 | 0.005 | 0.003 | 0.004 | 0.000 |  |  |
| *Loxocauda orientalis*_I41 | 0.003 | 0.003 | 0.005 | 0.005 | 0.003 | 0.004 | 0.000 | 0.000 |  |
| *Loxocauda orientalis*_I42 | 0.003 | 0.003 | 0.005 | 0.005 | 0.003 | 0.004 | 0.000 | 0.000 | 0.000 |
